# Prediction of EGFR Mutation Status in Non–Small Cell Lung Cancer Based on Ensemble Learning

**DOI:** 10.3389/fphar.2022.897597

**Published:** 2022-06-27

**Authors:** Youdan Feng, Fan Song, Peng Zhang, Guangda Fan, Tianyi Zhang, Xiangyu Zhao, Chenbin Ma, Yangyang Sun, Xiao Song, Huangsheng Pu, Fei Liu, Guanglei Zhang

**Affiliations:** ^1^ Beijing Advanced Innovation Center for Biomedical Engineering, School of Biological Science and Medical Engineering, Beihang University, Beijing, China; ^2^ School of Medical Imaging, Shanxi Medical University, Taiyuan, China; ^3^ College of Advanced Interdisciplinary Studies, National University of Defense Technology, Changsha, China; ^4^ Beijing Advanced Information and Industrial Technology Research Institute, Beijing Information Science and Technology University, Beijing, China

**Keywords:** non–small cell lung cancer, radiogenomics, EGFR, computed tomography, ensemble learning

## Abstract

**Objectives:** We aimed to identify whether ensemble learning can improve the performance of the epidermal growth factor receptor (EGFR) mutation status predicting model.

**Methods:** We retrospectively collected 168 patients with non–small cell lung cancer (NSCLC), who underwent both computed tomography (CT) examination and EGFR test. Using the radiomics features extracted from the CT images, an ensemble model was established with four individual classifiers: logistic regression (LR), support vector machine (SVM), random forest (RF), and extreme gradient boosting (XGBoost). The synthetic minority oversampling technique (SMOTE) was also used to decrease the influence of data imbalance. The performances of the predicting model were evaluated using the area under the curve (AUC).

**Results:** Based on the 26 radiomics features after feature selection, the SVM performed best (AUCs of 0.8634 and 0.7885 on the training and test sets, respectively) among four individual classifiers. The ensemble model of RF, XGBoost, and LR achieved the best performance (AUCs of 0.8465 and 0.8654 on the training and test sets, respectively).

**Conclusion:** Ensemble learning can improve the model performance in predicting the EGFR mutation status of patients with NSCLC, showing potential value in clinical practice.

## Introduction

According to the estimates of cancer burden by GLOBOCAN 2020, lung cancer remained the most common cancer type and the leading cause of cancer death in China (Cao et al., 2021). Non–small cell lung cancer (NSCLC) is the main type of lung cancer, which accounts for 80%–85% (Singh et al., 2021). In recent years, molecular targeted therapy has become one of the effective treatments in clinical tumor therapy for lung cancer. Studies have shown that some genetic markers contribute to prognosis remarkably, such as the epidermal growth factor receptor (EGFR), kirsten rat sarcoma viral oncogene (KRAS), and anaplastic lymphoma kinase (ALK) genes (Yang et al., 2017; Alanazi et al., 2020; Lee et al., 2021).

EGFR, a transmembrane glycoprotein, comprising 1,186 amino acids with tyrosine kinase (TK) activity, plays an important role in signal transduction. EGFR is encoded by the EGFR gene, which contains 28 exons. Mutations on its exons 18, 19, 20, and 21 may lead to abnormal EGFR activation (Herbst et al., 2018), which is one of the causes of tumorigenesis.

Many studies have found that EGFR gene mutation status is closely related to some EGFR-targeting drugs, such as tyrosine kinase inhibitor (TKI). In 2004, Paez et al. (2004) found that TKI had a significant effect on patients with EGFR mutations, which were more common in women, adenocarcinoma patients, non-smokers, and Asians. In addition, studies have shown that 75% of the patients with EGFR mutations respond better to TKI treatment than those without mutations (Gazdar, 2009). The current explanation for this phenomenon is that mutations in the EGFR gene enhance the sensitivity of tumor cells to TKIs. Therefore, EGFR mutation status is considered to be a predictor of the therapeutic effect of TKI.

At present, the gold standard of EGFR gene mutation status detection is DNA sequencing after tissue biopsy (Paez et al., 2004). However, tissue biopsy is difficult to be widely used in clinical practice due to its invasive nature, difficulties in repetition, and temporal and spatial heterogeneity of tumors. Therefore, it is necessary to find a noninvasive, easily repeatable, and comprehensive detection method. The emergence of radiomics makes it possible to solve this problem.

The concept of radiomics was first proposed by Lambin in 2012 (Lambin et al., 2012). Based on the development of machine learning and data mining technology, radiomics points out a medical image analysis methodology that combines image segmentation, feature extraction, feature analysis, and data mining. Radiomics has been widely used in medical image analysis, such as early screening and subtype classification of tumors and prediction of patient survival. Furthermore, radiogenomics combines radiomics with genomics to analyze the relationship between radiomics features in medical images and gene mutation status at the molecular level.

There has been a lot of research on EGFR mutation status prediction by radiomics (Gevaert et al., 2017; Velazquez et al., 2017; Jia et al., 2019; Jiang et al., 2019; Li et al., 2019; Pinheiro et al., 2020). Velazquez E R et al. (2017) established a random forest (RF) model with 16 radiomics features extracted from 763 patients, which obtained the area under the curve (AUC) of 0.8 on the test set. After combining radiomics features with semantic features, the AUC of the RF model was improved to 0.86. Pinheiro G et al. (2020) used principal component analysis (PCA) and t-distributed stochastic neighbor embedding (T-SNE) to select the features. The extreme gradient boosting (XGBoost) model established with selected features obtained the highest AUC of 0.83.

It can be concluded that the current research studies on EGFR mutation status prediction mostly used a single classifier and most of them evaluated the model performance only on one test set, making the results not convincing enough. In addition, the performances of previous models need to be improved to make the model available in clinical diagnosis. In this research, we tried to explore the relationships between CT radiomics features and EGFR mutation status in patients with NSCLC and construct a more effective model based on ensemble learning.

## Materials and Methods

### Patients

Our research is based on the public dataset NSCLC-Radio Genomics by the Cancer Imaging Archive (TCIA) (Bakr et al., 2018), including data from 211 patients with NSCLC from the Stanford University School of Medicine and the Alto Veterans Affairs Health Care System. The patients were selected from a pool of early-stage NSCLC patients, receiving CT examination within 1 month before surgery. The tissue slices from patients were later used to obtain mutation data and gene expression data using gene expression microarrays or RNA-sequencing or both. A total of 168 subjects (125 mutant type; 43 wild type) were finally enrolled in our study, excluding projects without the EGFR phenotype.

### CT Image Acquisition and EGFR Mutation Detection

CT images were recorded in DICOM format. Since this is a retrospectively collected dataset, different subjects were scanned using different scanners, scanning protocols, and scanning parameters. The common scanning parameters were as follows: slice thickness of 0.625–3 mm (median: 1.5 mm) and an X-ray tube current of 124–699 mA (mean 220 mA) at 80–140 kVp (mean 120 kVp). All CT image slice thicknesses were unified to 1 mm with an interpolation algorithm before segmentation. Other detailed parameters were recorded in the DICOM headers.

Mutation detection was performed on exons 18, 19, 20, and 21 on the EGFR gene in 206 patients, of which 125 patients were wild type, 43 patients were mutant, and the information of others was lost. Therefore, a total of 168 patients had the EGFR mutation status, which was stored in the CSV file of clinical information.

### Region of Interest Segmentation

Initial ROI segmentation of 144 subjects was provided by the TCIA with an unpublished automatic segmentation algorithm. After that, all segmentations were viewed and checked by a professional thoracic radiologist and an additional thoracic radiologist. The segmentations from the TCIA were incomplete, so we made further process.

The anonymized thin-slice CT images (1 mm, DICOM format) were delineated and segmented on lung window (window width, 1200 HU; window level, −500 HU) using ITK-SNAP (www.itk-snap.org). Two radiologists with 15 and 4 years of experience in chest CT image interpretation, blinded to the clinical data of each subject, segmented the nodules slice by slice. Finally, segmentation results were output as three-dimensional ROI files (NRRD format) for subsequent feature extraction.

### Feature Extraction

**FIGURE 1:**
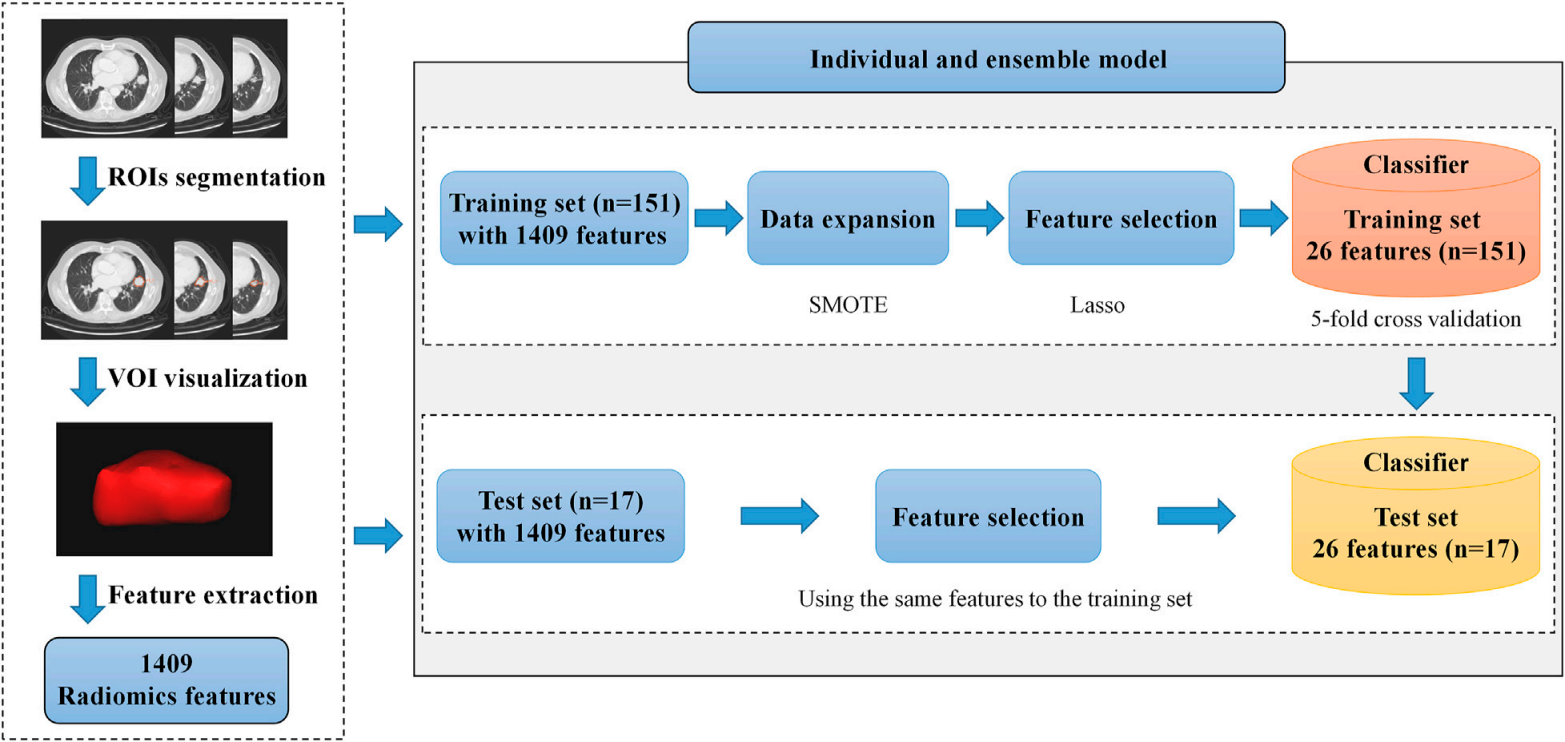
Framework of our proposed radiomics model. It includes volume of interest (VOI) segmentation, radiomics feature extraction, and model construction. In the model construction process, we make data expansion with the SMOTE on the training set and feature selection on the training and the test sets. The most appropriate hyperparameter of the model is selected by the average accuracy on the validation set in the training process, and the best model is sent to the testing process.

In this study, we extracted 1,409 radiomics features (Figure 1) from the three-dimensional volume of interest (VOI) for each subject by the pyradiomics package (vision 2.1.2). The extracted radiomics features mainly include three categories: shape, intensity, and texture.

The 14 shape features were calculated from the VOIs directly, reflecting the shape of the VOI region in two and three dimensions, describing the size and shape of VOI, such as elongation, flatness, surface area to volume ratio, and volume. In addition to the original image, 14 filters were used to obtain additional information and both the original VOIs and filtered VOIs were calculated in the process of intensity feature and texture feature extraction. 18 of the 270 intensity features were extracted from the original VOIs, and the others were extracted from the filtered VOIs. Intensity features can reflect the intensity distribution of voxel points in the image, including entropy, energy, maximum, minimum, average, and median. Similar to the intensity features, 1,125 texture features were extracted through five matrixes on the original and filtered VOIs. Texture features describe the texture of an image with gray changes, such as autocorrelation, average intensity, energy, contrast, and gray difference (Sacconi et al., 2017; Takeda et al., 2017).

### Data Division and Expansion

The extracted radiomics features were divided into the training set and test set in the ratio of 9:1, shown in Table 1. In this research, each subject is obtained only from one patient, so there is no risk of data leakage. Due to the imbalance between the two categories (the number of wild types is three times as common as mutant types), we used the synthetic minority oversampling technique (SMOTE) algorithm (Chawla et al., 2002) only on the training set to balance the dataset. After the SMOTE, the number of subjects in both categories is equal.

**TABLE 1:** Number of each phenotype in the dataset before the SMOTE.

	Wild type	Mutant type	Total
Training set	112	39	151
Test set	13	4	17
Total	125	43	168

### Feature Selection

In this research, a total of 1,409 features were extracted, whose number was much larger than that of patients, causing the overfitting of the model. To reduce the dimension of radiomics features, this research used the variance selection method and Lasso algorithm (Robert, 2018) for feature selection after data normalization on Python 3.7. In addition, z-score was used for standardization to make features conform to normal distribution before feature selection.

### Model Construction

**FIGURE 2:**
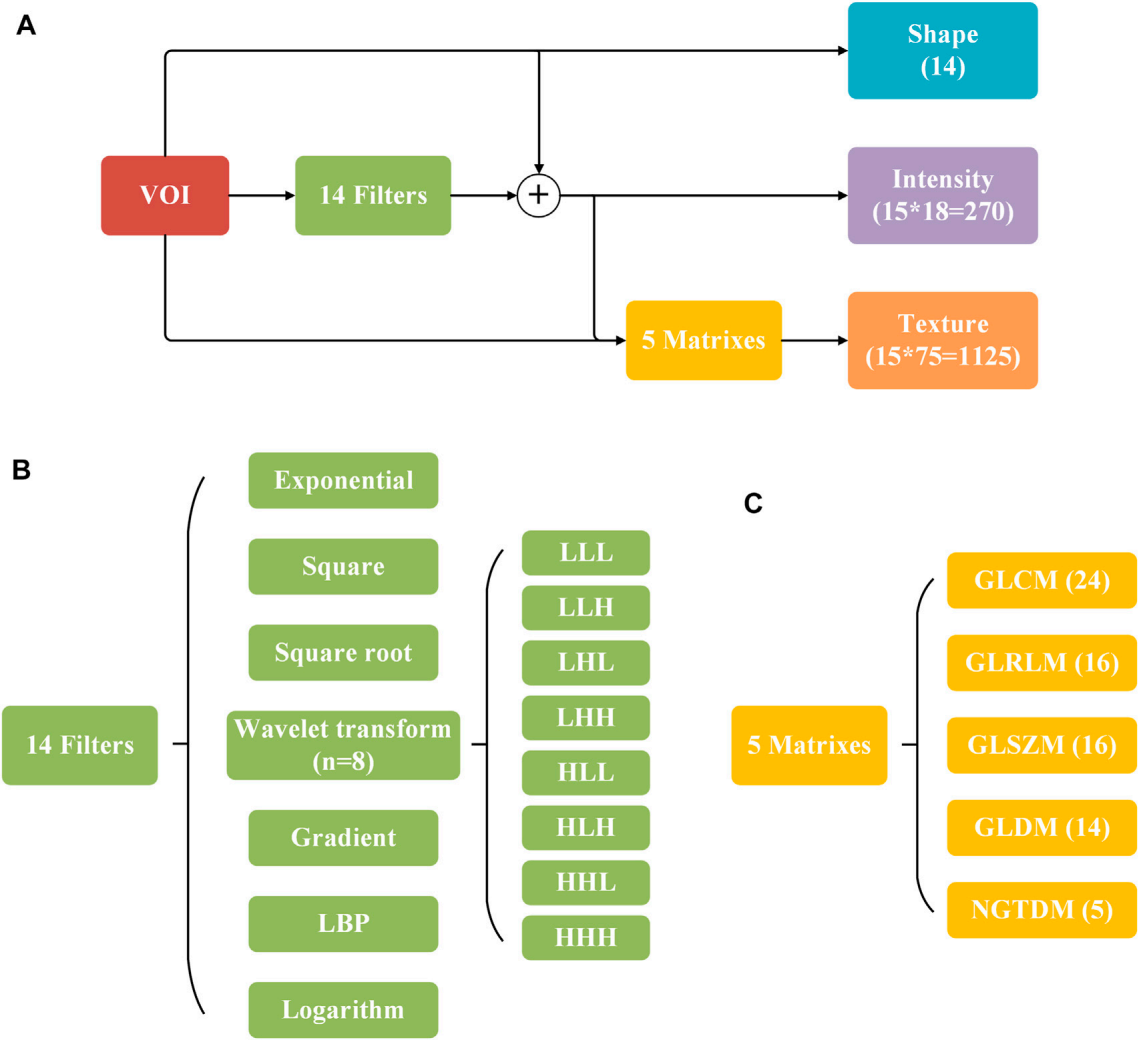
Description of 1,409 radiomics features. **(A)** Process of feature extraction. **(B,C)** 14 filters and five matrixes used in **(A)**. The numbers of extracted features are indicated in parentheses.

After feature selection, the selected features were sent into the prediction model. First, we used individual classifiers independently as prediction models, including RF (Breiman, 2001), XGBoost (Chen and Guestrin, 2016), logistic regression (LR) (Kleinbaum and Klein, 2010), and support vector machine (SVM) (Cortes and Vapnik, 1995). After that, we used two ensemble learning strategies, hard voting and soft voting, to combine the advantages of individual classifiers. Furthermore, to obtain the optimal hyperparameters of models, we used 5-fold cross-validation on the training set and selected hyperparameters according to the average accuracy of models on the five validation sets. The strategies of individual and ensemble model construction are summarized in Figure 2.

## Result

### Results of Feature Selection

After feature extraction, 1,409 radiomics features were obtained from the original dataset. The process of feature selection used variance selection and the Lasso algorithm successively. In variance selection, the threshold value was set to 0, meaning the unchanged features were filtered out. There were 1,243 original features left through the variance filter.

**FIGURE 3:**
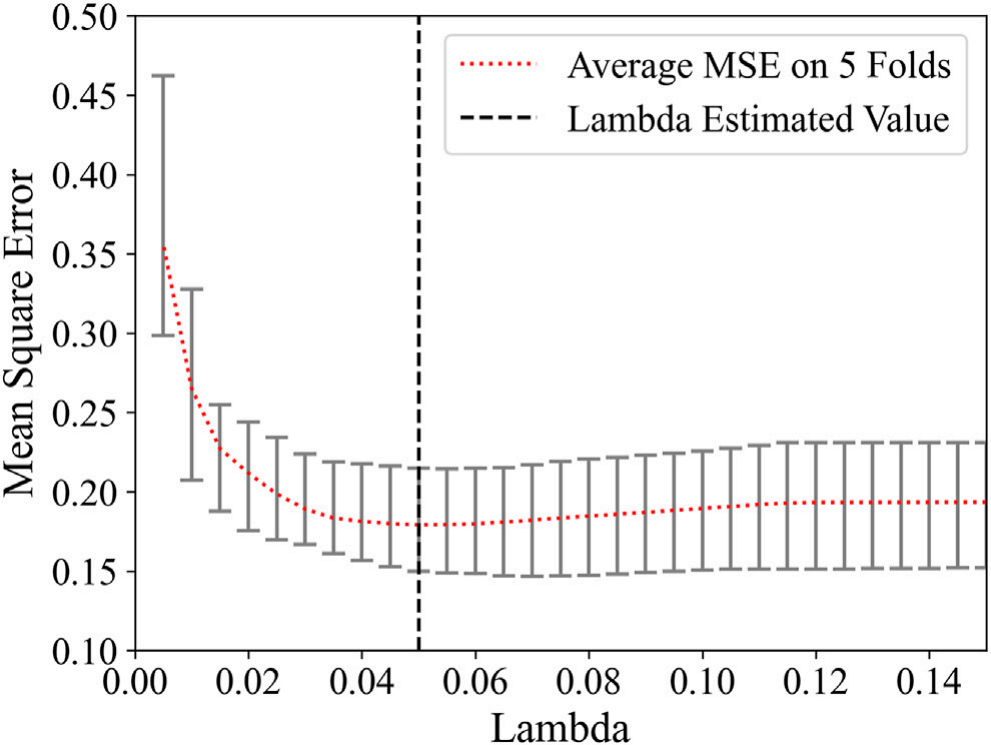
Feature selection using the LASSO method. Relationship of MSE and *λ*.

For a great influence on the feature selection result of the regularization parameter *λ* in Lasso, a linear regression classifier was used with 5-fold cross-validation on the training set to obtain the appropriate value of *λ*. The relationship between loss function MSE and *λ* is shown in Figure 3. Marked by the black vertical line, when the value of *λ* is 0.05, the average MSE on five-folds is the lowest.

**FIGURE 4:**
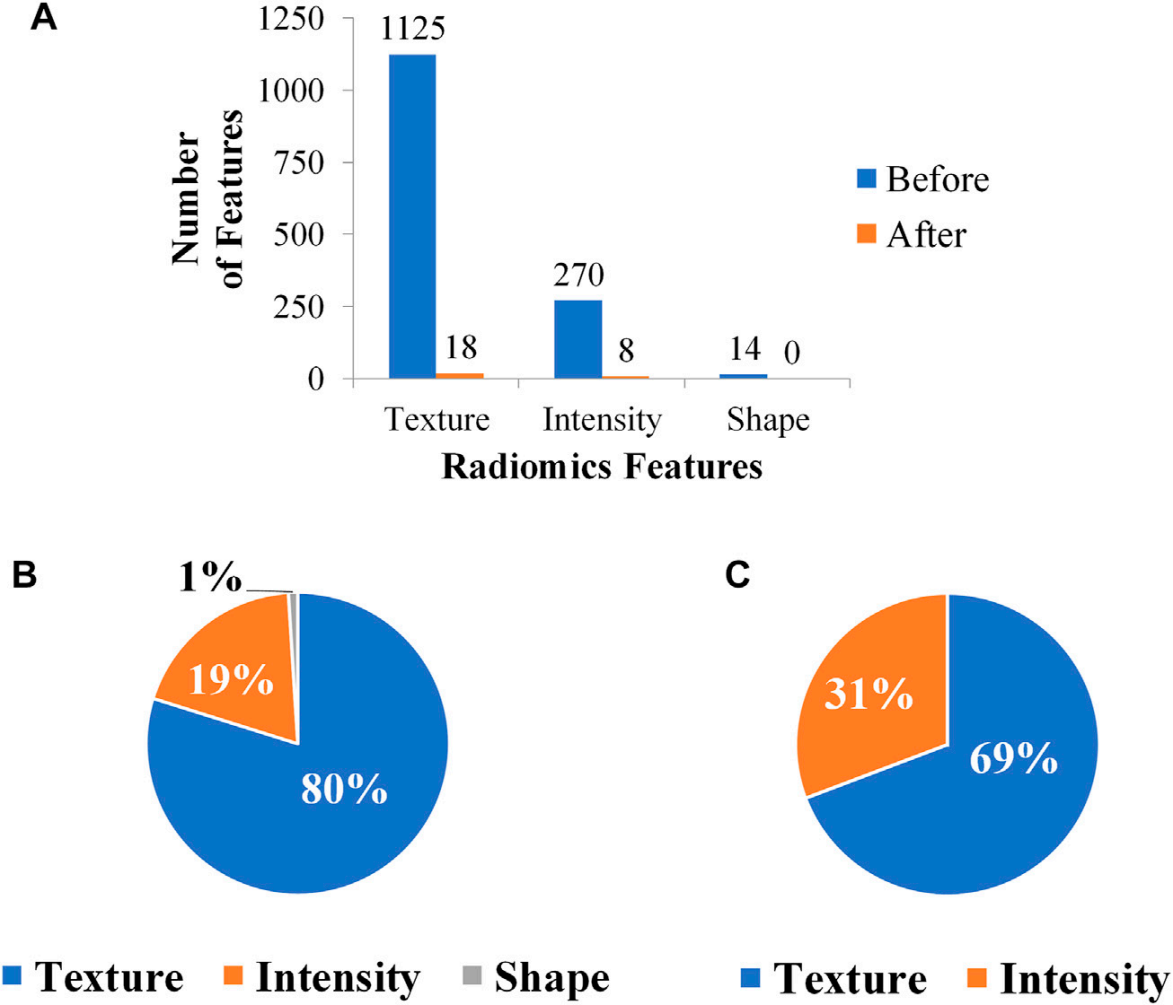
Variation of feature number through feature selection. **(A)** Feature number before and after feature selection. **(B)** Feature ratio before feature selection. **(C)** Feature ratio after feature selection.

**FIGURE 5:**
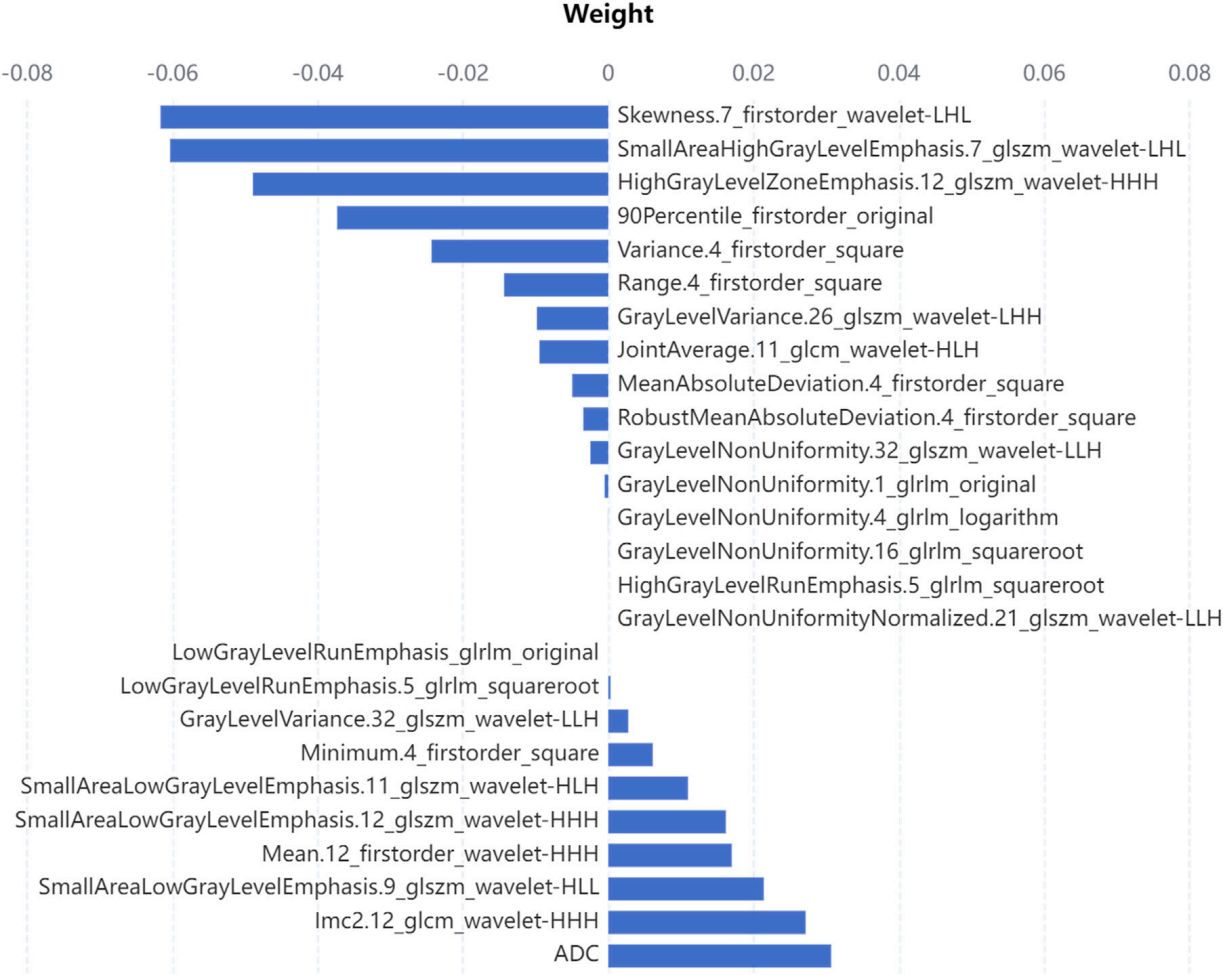
Weight of selected features with Lasso in the linear regression classifier.

Searching around 0.05, the *λ* was eventually determined to be 0.04. There were 26 remaining features after feature selection, and the number of each category was compared in Figure 4. The proportion of intensity features increases after feature selection, so it is speculated that intensity features have a great contribution to this research; on the contrary, shape features may contribute little to this research for its number is 0 after feature selection. The weight of each selected feature in the linear regression classifier is shown in Figure 5. It can be seen that skewness and small area high gray level emphasis contribute most to this model.

### Performance of Individual Models

In this research, the selected features were input into four individual classifiers for training. During the training process, the optimal hyperparameters were determined according to the performance of the individual classifier on the validation set. Then, each individual classifier was trained with all data on the training set, and the obtained individual model was used to make a prediction on the test set. The performance of each individual classifier on the validation set and test set is shown in Table 2.

**TABLE 2:** ACC and AUC of individual models.

Classifier	Validation set		Test set	
	ACC (Mean ± Std)	AUC (Mean ± Std)	ACC	AUC
LR	0.7944 ± 0.0542	0.8607 ± 0.0547	0.7647	0.7885
SVM	0.7942 ± 0.0503	0.8634 ± 0.0517	0.7647	0.7885
RF	0.7744 ± 0.069	0.7815 ± 0.0741	0.7647	0.8269
XGBoost	0.7744 ± 0.078	0.7911 ± 0.1129	0.7647	0.8269

As displayed in Table 2, the LR and SVM obtain the highest average accuracy and average AUC, respectively, in the 5-fold cross-validation. For the SVM, the standard deviations of accuracy and AUC are the smallest, which demonstrates that the SVM has stronger robustness than the others for its less impact caused by data disturbance. On the test set, the accuracies of the four classifiers are same, among which RF and XGBoost have the highest AUC, indicating that ensemble learning can improve the generalization performance of the classifier.

**FIGURE 6:**
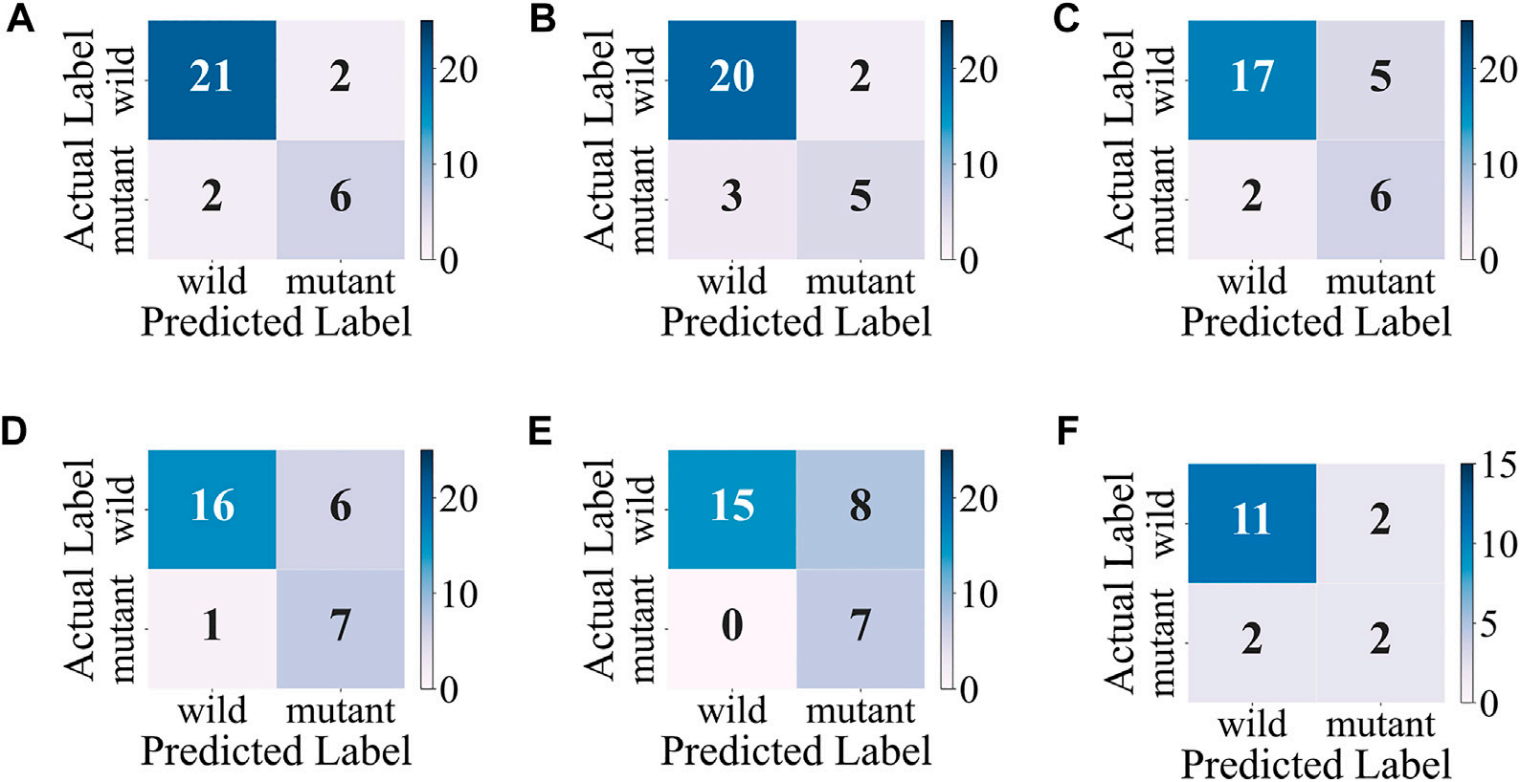
Confusion matrixes of the SVM. **(A–E)** Confusion matrixes on the validation sets during the 5-fold cross-validation. **(F)** Confusion matrix on the test set.

Considering the accuracy and stability of the model, the SVM obtains the best overall performance among individual classifiers. As shown in Figure 6, the SVM makes a good classification for the small category (mutant type), but the classification results for the major category (wild type) are not ideal, with a high false-positive rate. At the same time, the model classification results on the test set are slightly inferior to those on the validation set, so further improvement for the model is needed.

### Performance of Ensemble Models

Three or four individual classifiers were selected for both hard-voting and soft-voting. We evaluated the performance of ensemble models with accuracy and AUC on the validation set and test set and part of which is shown in Table 3.

**TABLE 3:** ACC and AUC of ensemble models.

Classifier	Voting method	Validation set		Test set	
		ACC (Mean ± Std)	AUC (Mean ± Std)	ACC	AUC
LR	—	0.7944 ± 0.0542	0.8607 ± 0.0547	0.7647	0.7885
SVM	—	0.7942 ± 0.0503	0.8634 ± 0.0517	0.7647	0.7885
RF	—	0.7744 ± 0.069	0.7815 ± 0.0741	0.7647	0.8269
XGBoost	—	0.7744 ± 0.078	0.7911 ± 0.1129	0.7647	0.8269
RF + XGBoost + LR	soft	0.7944 ± 0.0653	0.8465 ± 0.0659	0.8824	0.8654
XGBoost + SVM + LR	soft	**0.8275 ± 0.0264**	**0.8632 ± 0.0559**	0.8235	0.8462
RF + XGBoost + SVM	soft	0.8011 ± 0.0480	0.8453 ± 0.0684	**0.8235**	**0.8654**
RF + XGBoost + LR	hard	0.7811 ± 0.0695	—	0.8235	—
All	hard	0.8211 ± 0.0456	—	0.7647	—
all	soft	0.8144 ± 0.0275	0.8587 ± 0.0550	0.7647	0.8654

The best performance in the models is highlighted in bold.

It can be seen that the performance of the ensemble classifier is better than that of the individual classifier. The combination of RF, XGBoost, and LR obtains the best performance on the test set, while it is not satisfactory on the validation set. Considering the model performance on the validation set, the combination of XGBoost, SVM and LR has the best comprehensive performance and the model is more robust, with the further performance on the test set shown in Table 4.

**TABLE 4:** Further performance of the combination of XGBoost, SVM, and LR on the test set.

Classifier	Accuracy	Precision	Recall	F1-score
SVM	0.76	0.67	0.67	0.67
LR	0.76	0.70	0.76	0.72
XGBoost	0.76	0.75	0.85	0.74
Hard-voting	0.76	0.70	0.76	0.72
Soft-voting	0.82	0.76	0.80	0.77

**FIGURE 7:**
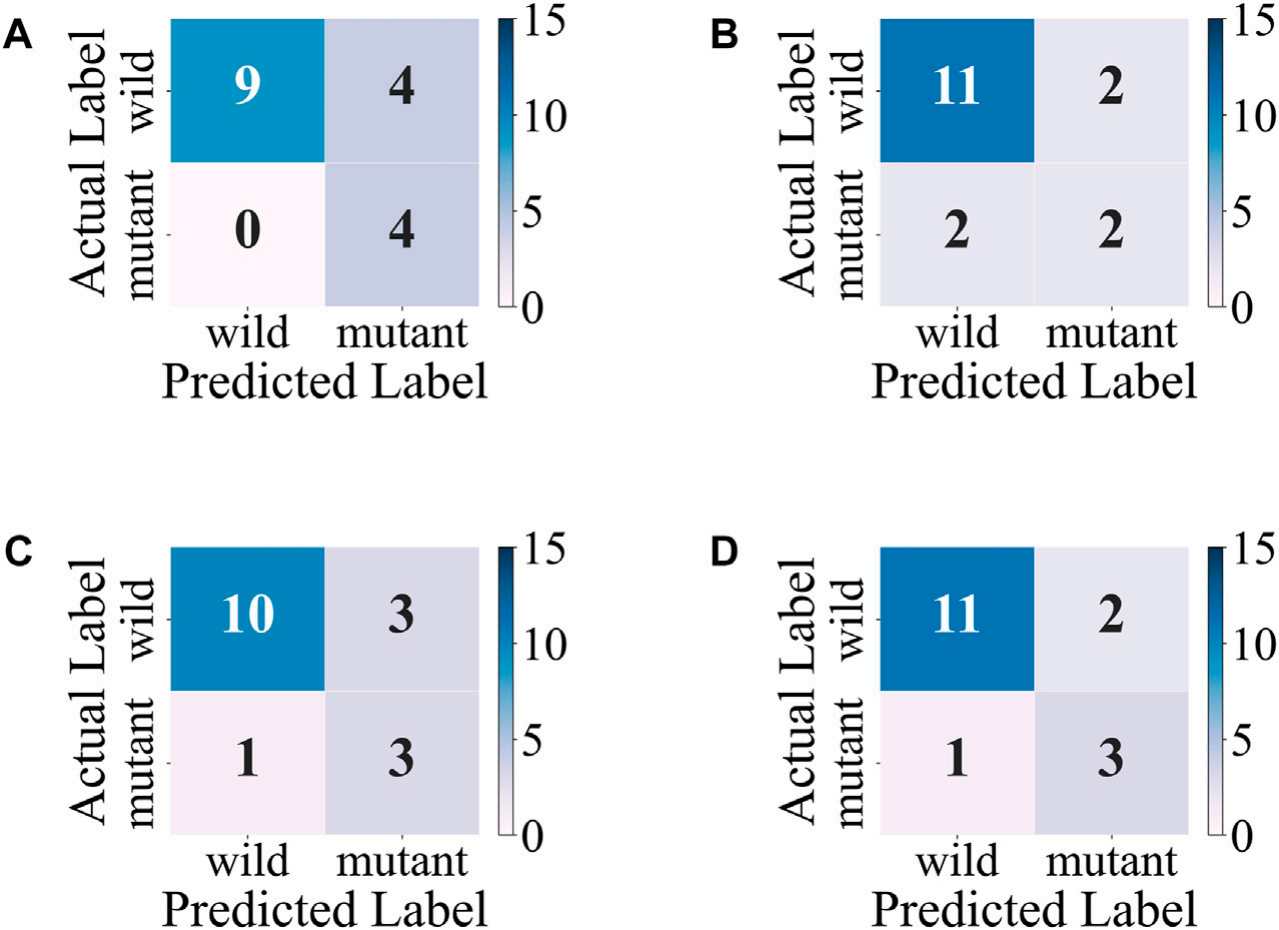
Confusion matrixes of the ensemble model and individual models on the test set. **(A)** XGBoost. **(B)** SVM. **(C)** LR. **(D)** Ensemble model with soft-voting of XGBoost, SVM, and LR.

**FIGURE 8:**
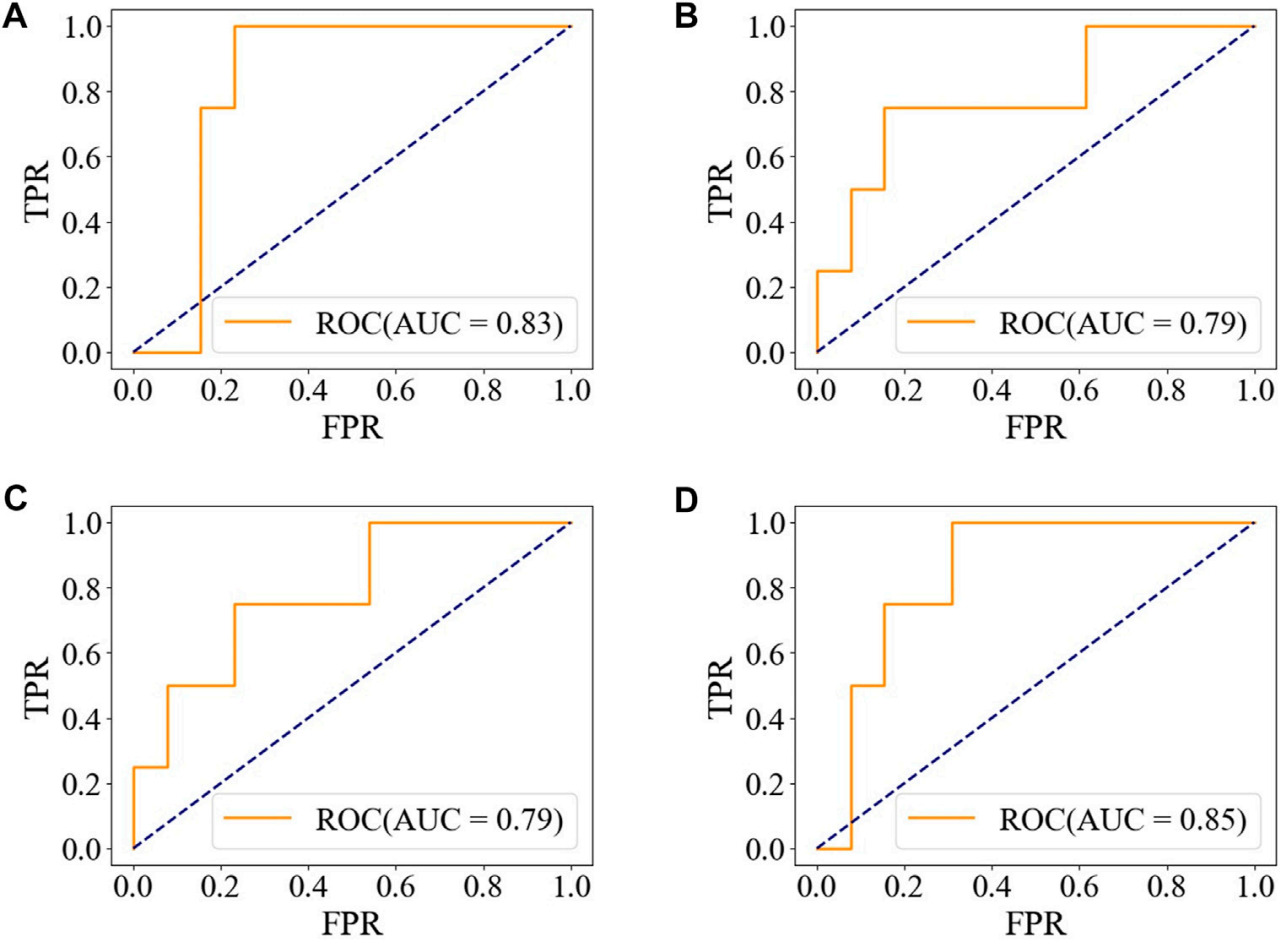
ROC of the ensemble model and individual models on the test set. **(A)** XGBoost. **(B)** SVM. **(C)** LR. **(D)** Ensemble model with soft-voting of XGBoost, SVM, and LR.

The accuracy, precision, and F1-score of the ensemble model with soft-voting of XGBoost, SVM, and LR are improved compared with those of individual classifiers, and even the recall is slightly lower than that of XGBoost but is higher than that of SVM and LR, proving that ensemble learning can combine the advantages of individual classifier and obtain better performance. Compared with individual classifiers through confusion matrix (Figure 7) and ROC curve (Figure 8), the false-positive rate of the ensemble model is significantly reduced, which means the ensemble model predicts better on the major category.

## Discussion

Computer-aided diagnosis has shown great potential in many fields. Radiogenomics is one of the fields in computer-aided diagnosis, combining radiomics with genomics using high-throughput radiomics features extracted from medical images to explore molecular information, such as gene mutation status. The exploration of radiogenomics can not only work in early diagnosis in improving the survival rate of a certain disease but also provide clues to the physiological mechanisms at the molecular level.

Based on 1,409 radiomics features extracted from the CT images of NSCLC patients, this research constructed many models with individual classifiers and ensemble classifiers for predicting the EGFR mutation status. When the individual classifier was used alone, each classifier showed a different advantage. SVM performed best among them for its outstanding robustness with lowest variance, and LR achieved an average performance on the validation sets and test set. As for two classifiers based on ensemble learning, RF obtained smaller variance on accuracy and AUC, while XGBoost obtained a higher average, supporting that bagging ensemble can reduce variance while boosting ensemble reduces bias. Compared with the SVM, RF and XGBoost showed better generalization ability on the test set.

To combine the advantages of individual classifiers and find a better ensemble strategy, we tried different combinations of three or four classifiers with different voting methods. As Table 3 shows, the highest average accuracy and minimum variance on the validation set appear on the combination of XGBoost, SVM, and LR with soft-voting. Compared to the individual classifier, the ensemble model improved the accuracy value by nearly three percent and significantly reduced the variance. This combination absorbed the high accuracy of LR and low variance of the SVM, overcoming the instability of XGBoost, which showed advantages of ensemble learning. In conclusion, it was speculated that the combination of these three classifiers plays a complementary role in this research. As for the impact of the voting method, classifiers using soft-voting performed better than hard-voting because soft-voting is based on probability produced by the classifier, reducing the impact on classification errors by the individual classifier, while hard-voting is based on 0 or 1.

Compared with individual models, the performance of ensemble models was improved on almost all indexes, and the false-positive rate was significantly reduced. Our ensemble model achieved the highest accuracy of 0.88 and the AUC of 0.86 on the test set, exceeding the level of current other studies, which indicated that the ensemble learning method was effective.

However, there are still some limitations in this research: 1) the gold standard for obtaining EGFR mutation status is DNA sequencing after tissue biopsy, which means it is difficult to build a large dataset for model construction; 2) this research is only based on radiomics features, so the combination with semantic features may further improve the model performance according to previous studies (Jia et al., 2019; Jiang et al., 2019; Li et al., 2019).

In conclusion, this research explored the effectiveness of ensemble learning in predicting the EGFR mutation status, showing that ensemble learning can improve the model’s accuracy and robustness. In addition, compared with shape features, intensity features may play a more important role in EGFR mutation prediction. For further research, we will try to build a larger dataset and construct the model with both semantic features and radiomics features.

## Data Availability

The original contributions presented in the study are included in the article/Supplementary Material, further inquiries can be directed to the corresponding author.
